# The Use of Dietary Approaches to Stop Hypertension (DASH) Mobile Apps for Supporting a Healthy Diet and Controlling Hypertension in Adults: Systematic Review

**DOI:** 10.2196/35876

**Published:** 2022-11-02

**Authors:** Ghadah Alnooh, Tourkiah Alessa, Mark Hawley, Luc de Witte

**Affiliations:** 1 Centre for Assistive Technology and Connected Healthcare School of Health and Related Research University of Sheffield Sheffield United Kingdom; 2 Department of Health Sciences College of Health and Rehabilitation Sciences Princess Nourah Bint Abdulrahman University Riyadh Saudi Arabia; 3 Biomedical Technology Department College of Applied Medical Sciences King Saud University Riyadh Saudi Arabia

**Keywords:** DASH diet, Dietary Approaches to Stop Hypertension, smartphone app, mobile app, blood pressure

## Abstract

**Background:**

Uncontrolled hypertension is a public health issue, with increasing prevalence worldwide. The Dietary Approaches to Stop Hypertension (DASH) diet is one of the most effective dietary approaches for lowering blood pressure (BP). Dietary mobile apps have gained popularity and are being used to support DASH diet self-management, aiming to improve DASH diet adherence and thus lower BP.

**Objective:**

This systematic review aimed to assess the effectiveness of smartphone apps that support self-management to improve DASH diet adherence and consequently reduce BP. A secondary aim was to assess engagement, satisfaction, acceptance, and usability related to DASH mobile app use.

**Methods:**

The Embase (OVID), Cochrane Library, CINAHL, Web of Science, Scopus, and Google Scholar electronic databases were used to conduct systematic searches for studies conducted between 2008 and 2021 that used DASH smartphone apps to support self-management. The reference lists of the included articles were also checked. Studies were eligible if they (1) were randomized controlled trials (RCTs) or pre-post studies of app-based interventions for adults (aged 18 years or above) with prehypertension or hypertension, without consideration of gender or sociodemographic characteristics; (2) used mobile phone apps alone or combined with another component, such as communication with others; (3) used or did not use any comparator; and (4) had the primary outcome measures of BP level and adherence to the DASH diet. For eligible studies, data were extracted and outcomes were organized into logical categories, including clinical outcomes (eg, systolic BP, diastolic BP, and weight loss), DASH diet adherence, app usability and acceptability, and user engagement and satisfaction. The quality of the studies was evaluated using the Cochrane Collaboration’s Risk of Bias tool for RCTs, and nonrandomized quantitative studies were evaluated using a tool provided by the US National Institutes of Health.

**Results:**

A total of 5 studies (3 RCTs and 2 pre-post studies) including 334 participants examined DASH mobile apps. All studies found a positive trend related to the use of DASH smartphone apps, but the 3 RCTs had a high risk of bias. One pre-post study had a high risk of bias, while the other had a low risk. As a consequence, no firm conclusions could be drawn regarding the effectiveness of DASH smartphone apps for increasing DASH diet adherence and lowering BP. All the apps appeared to be acceptable and easy to use.

**Conclusions:**

There is weak emerging evidence of a positive effect of using DASH smartphone apps for supporting self-management to improve DASH diet adherence and consequently lower BP. Further research is needed to provide high-quality evidence that can determine the effectiveness of DASH smartphone apps.

## Introduction

### Background

Hypertension is a serious medical condition that has become a public health problem. Globally, in 2015, 1.13 billion people (1 out of 4 men and 1 out of 5 women) had hypertension, and most of them were living in low- and middle-income countries [1]. Hypertension is attributed to the following 2 kinds of risk factors: (1) modifiable risk factors, which include unhealthy diet, physical inactivity, obesity, and consumption of tobacco and alcohol; and (2) nonmodifiable risk factors, which include family history of hypertension, age over 65 years, and chronic diseases, such as diabetes and kidney disease [1]. Uncontrolled hypertension might lead to significant complications, such as heart failure, stroke, kidney failure, and economic difficulties stemming from both treatment costs and human capital loss [2-6]. Several studies have shown that hypertension is often poorly controlled and that treatment measures include preventive behaviors and risk factor management [2,5,7]. The World Health Organization recommends the participation of patients through self-monitoring of weight, consumption of diets that are low in sodium and fat, physical activity, smoking cessation, stress reduction, and regular hospital visits to better control hypertension [6].

Self-management is one of the most effective approaches for dealing with hypertension, allowing people with hypertension to feel more responsible for their own health [8]. The Joint National Committee on the Prevention, Detection, Evaluation, and Treatment of High Blood Pressure has given 6 self-management recommendations that are considered essential for high blood pressure (BP) control: (1) adhering to medication protocols, (2) following the Dietary Approaches to Stop Hypertension (DASH) diet, (3) engaging in physical activities, (4) limiting alcohol consumption, (5) avoiding tobacco, and (6) maintaining a healthy weight [9].

The DASH diet was established by the National Heart, Lung, and Blood Institute (NHLBI) [10]. It provides basic recommendations for a balanced healthy diet that includes various foods [11]. Specifically, the DASH diet comprises vegetables, fruits, whole grains, fish, poultry, beans, nuts, and healthy oils [12]. The DASH diet also recommends a sodium intake of 2300 mg/day or 1500 mg/day for high-risk individuals (eg, those with hypertension or type 2 diabetes) [12]. The diet is also focused on consuming foods that are rich in potassium, calcium, magnesium, protein, and fiber [12].

Consumption of the DASH diet is correlated with a reduction in BP [12]. Recently, an umbrella review was conducted to summarize the available systematic reviews and meta-analyses of randomized controlled trials (RCTs) on different dietary patterns that reduce BP [13]. The review found that a decline in BP correlated with the DASH diet, with the mean differences ranging from −3.20 mmHg to −7.62 mmHg for systolic blood pressure (SBP) and from −2.50 mmHg to −4.22 mmHg for diastolic blood pressure (DBP) [13]. In addition, for 8 years in a row, *US News and World Report* ranked the DASH diet developed by the National Institutes of Health as the “best overall” diet among almost 40 diets that were reviewed [14].

Additionally, systematic reviews have concluded that the DASH diet is beneficial for not only reducing BP, which was its original intended purpose, but also decreasing the risk of cardiovascular diseases, including that of the main subclasses “coronary heart disease,” “heart failure,” and “stroke” [15,16]. Furthermore, several systematic reviews investigating the DASH diet’s effects on insulin resistance and obesity have found that it may play an important role in controlling hyperglycemia and reducing weight [17,18]. Based on these results, the DASH diet has been promoted as a first-line nonpharmacological therapy along with lifestyle modifications for the treatment of many chronic diseases [15-18].

According to the NHLBI, adherence to the DASH diet in the United Sates is low [19]. Understanding the determinants of adherence is crucial for improving adherence [19]. At the clinical level, primary care physicians can offer guidance on proper nutritional habits for the treatment of hypertension [19]; however, physicians often state that they have insufficient time, resources, and knowledge for dietary counseling [19]. Additionally, commitment to several consulting sessions is challenging for patients [20].

Over the past decades, there has been a rapid increase in the use of smartphones, and by 2022, it is projected that there will be 6.8 billion smartphone users [21]. In parallel, there has been a rapid increase in mobile apps providing information and health services [21]. Smartphones running health apps are of particular interest because they can promote patient engagement and self-management, and allow for remote follow-up without the need for in-person physician visits [20,22,23].

### Aim

This review aimed at synthesizing existing evidence on the effectiveness of smartphone apps that support self-management to improve DASH diet adherence and accordingly reduce BP, as well as assessing app usability and acceptability, and user engagement and satisfaction. To the best of our knowledge, no studies have summarized the effects of DASH smartphone apps on DASH diet adherence.

## Methods

### Guideline

The Preferred Reporting Items for Systematic Reviews and Meta-Analyses (PRISMA) guideline for systematic reviews was used to conduct and report this systematic review [24].

### Data Sources and Search Methods

The following electronic databases were searched: Embase (OVID), Cochrane Library, CINAHL, Web of Science, Scopus, and Google Scholar. The databases were searched using keywords related to dietary approaches to stop hypertension, the DASH diet, and smartphone apps, and using MeSH terms, as well as appropriate synonyms (see Multimedia Appendix 1 for the search strategy). The terms were combined using Boolean operators OR and AND. The search was restricted to English language research published from 2008, when the first app store was introduced [25], to February 22, 2021. Google Scholar was used to search for any additional grey literature using a collection of text words chosen from the papers found in the electronic databases, such as “DASH diet mobile phone apps” and “DASH diet smartphone apps.” Reference lists of the included studies were checked by hand searching to find additional potentially relevant research.

### Inclusion Criteria

The population, intervention, comparison, outcome, and study design (PICOS) framework was used to create the inclusion criteria [24].

#### Population

The review included studies that involved people with prehypertension and hypertension who were aged 18 years or over, without consideration of gender or sociodemographic characteristics. Overweight and obese people, including those with hypertension, were included because a higher BMI is associated with a higher risk of eventually developing hypertension [26].

#### Intervention

The intervention target was mobile phone apps for dietary behavioral change. To be included, a study had to focus mainly on evaluating a mobile app that assists users in adopting, improving, or maintaining the DASH diet to reduce BP. Studies that combined the mobile phone app with another component, such as communication with others (eg, a coach or research team) by phone, text message, or email, were also included.

#### Comparator

The review included studies that used any comparator, for example, studies comparing usual care with the DASH mobile phone app or any other control intervention. Studies without a comparator, such as pre-post pilot studies, were also included.

#### Outcome

The primary outcome measures of the included studies were BP level and adherence to the DASH diet.

#### Study Design

This review included RCTs and pre-post studies. To minimize the probability of missing important articles both peer-reviewed articles and under-review articles were included.

### Exclusion Criteria

The following criteria were used to exclude studies: (1) Studies that focused on a healthy population, adolescents and children, or pregnant women; (2) Studies that only used messaging, which included text messaging, SMS text messaging, and emails, or only used websites; (3) Studies solely describing the development of a mobile system’s technology; (4) Studies that did not focus on the DASH diet; and (5) Conference abstracts, conference papers, protocols, and studies not published in English.

### Selection of Studies

Reference management software (Endnote X9.0, Clarivate Analytics) was used to import and collect study citations for selection and to deduplicate articles. The screening and selection of titles of studies were conducted by 2 researchers (GA and TA) independently based on eligible criteria. In the second phase, GA and TA checked the abstracts of selected titles. Titles and abstracts received 2 points if they matched the criteria, 0 points if they did not, and 1 point if there was doubt. A study was included in the next phase if the sum of reviewer scores for the title was 2 or more. Studies that received less than 2 points were excluded. Cohen kappa was used to evaluate the agreement of reviewers in each phase of the title and abstract selection process. Controversial studies and disagreements between reviewers were discussed with other researchers (LdW and MH).

### Data Extraction

Two reviewers (GA and TA) independently extracted data and cross-checked the data. The reviewers piloted a standardized form that was used to extract data. Any disagreements were resolved through discussion with the other researchers (LdW and MH) until consensus was obtained. The data included study characteristics (authors, year of publication, follow-up duration, and country); information on participants (sample size, age, gender, and diseases they had); information on apps (name, type, and functionalities); app input (information obtained from users and mode of entering user data); intervention characteristics; mode of intervention delivery (eg, stand-alone app or combined with another component such as phone or text message); and intervention content (information that the intervention gives to users). In addition, the theoretical framework used to develop or guide the intervention was extracted. For health outcomes, the primary and secondary health outcomes were extracted. We extracted the outcome data from the last follow-up and from both control and intervention groups.

### Data Analysis and Synthesis

A narrative summary of the studies was conducted. The data from each study were extracted, and the outcomes were organized into logical categories, including clinical outcomes (eg, SBP, DBP, and weight loss), DASH diet adherence, app usability and acceptability, and user engagement and satisfaction. The variety of study methods and reported outcomes meant that a meta-analysis was not possible. This review followed the PRISMA 2020 statement (Multimedia Appendix 2) [27].

### Assessment of the Risk of Bias

Two reviewers (GA and TA) independently assessed the risk of bias of the included studies. Any disagreements were addressed through discussion with the other researchers (LdW and MH). The risk of bias was assessed using the Cochrane Collaboration’s Risk of Bias Tool for RCTs, 2018 [28]. Risk ratings of “low,” “high,” and “some concerns” were assigned to the RCTs based on the presence of the following items: performance bias, selection bias, detection bias, attrition bias, reporting bias, and other bias. The overall risk of bias was high if any element was classified as high risk [28]. Robvis software, a visualization tool for risk of bias assessments in systematic reviews, was used [29].

Nonrandomized quantitative studies were evaluated using a tool for pre-post studies without a control group, which was provided by the US National Institute of Health (NIH) [30]. The quality ratings were “good,” “fair,” and “poor.” If the rating was poor, reasons were noted.

## Results

### Summary of the Search Results

**Figure 1:**
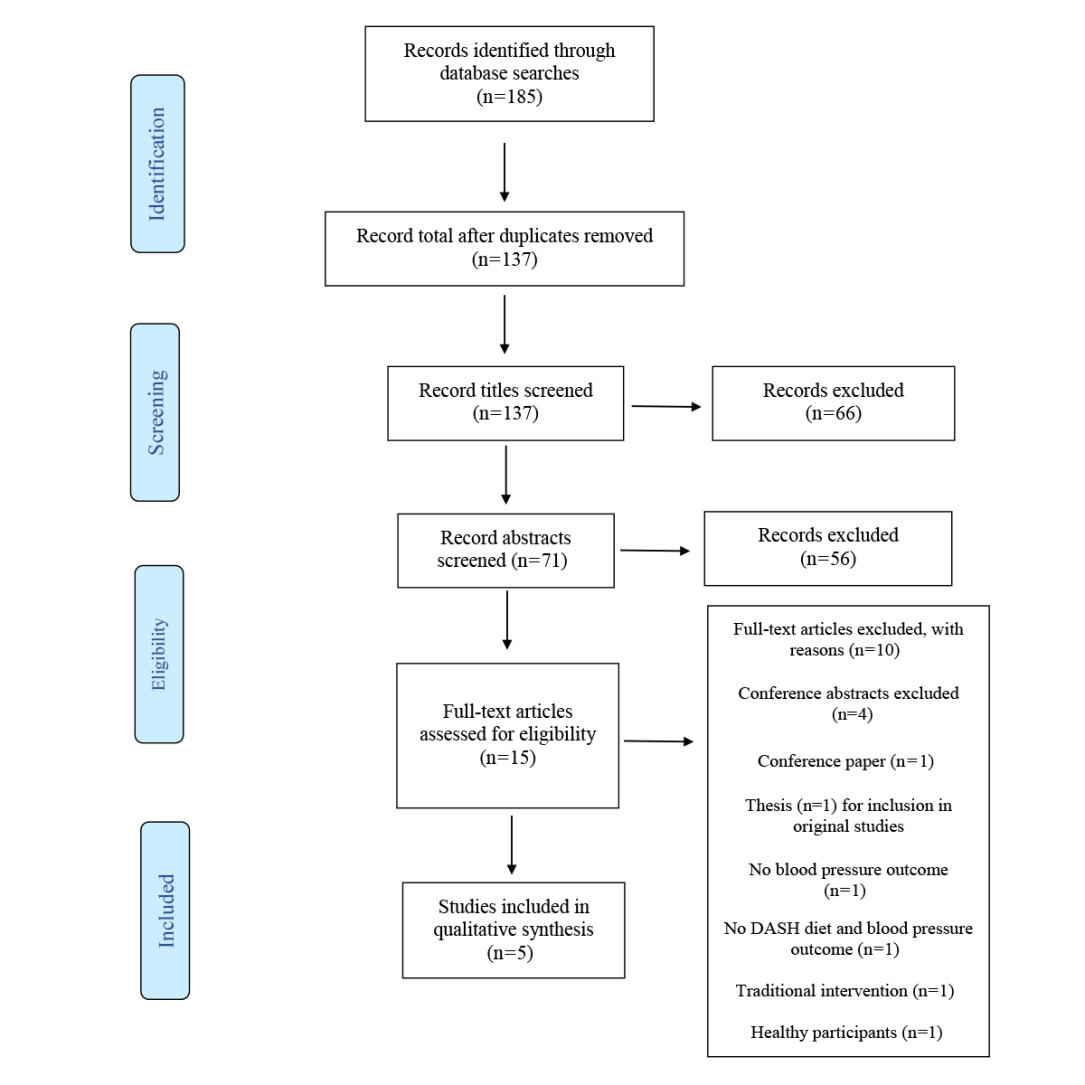
PRISMA (Preferred Reporting Items for Systematic Reviews and Meta-Analyses) flow diagram. DASH: Dietary Approaches to Stop Hypertension.

A total of 185 publications were identified from the searches, all of which came from electronic databases, as follows: 30 publications from Embase, 84 from Cochrane Library, 15 from CINAHL, 19 from Web of Science, 35 from Scopus, and 2 from Google Scholar. After duplicates were removed, 137 publications were screened for eligibility. From these, 122 were excluded after screening the titles and abstracts, and 15 full texts were retrieved. Examining the latter led to the exclusion of 10 publications that did not meet the inclusion criteria. In total, 5 publications were included in the analysis (Figure 1).

### Characteristics of the Studies

Of the 5 included studies, 3 were conducted in the United States [31-33] and 2 in Iran [34,35]. All were published between 2017 and 2021 (Multimedia Appendix 3). The included studies had sample sizes ranging from 17 to 120 participants, with a total of 334 participants. Four studies included both males and females [31,32,34,35], whereas 1 study included only females [33]. Participants from all the studies ranged in age from 18 to 75 years. Three studies [31,33,35] included participants with either hypertension or prehypertension alone, or participants with hypertension who were overweight or obese [32,34].

Of the 5 studies, 3 were RCTs [33-35] and 2 were pre-post pilot studies [31,32]. In terms of duration, the interventions were commonly conducted for 3 to 6 months [31-35].

All studies supported self-management of the DASH diet and hypertension. They all aimed to enhance self-management with increased patient awareness through educational information [31-35]. One study enhanced self-management without involving a human coach to monitor patients remotely [35], whereas the remaining 4 studies aimed to enhance self-management by involving a human coach [31,32] or research team to monitor patient data and health status remotely [33,34]. All studies reported the effectiveness of the apps in terms of dietary behavioral changes and controlling BP [31-35]. Four studies evaluated user engagement [31-33,35]; 2 assessed user satisfaction [33,35]; 1 evaluated acceptance [31]; 1 assessed usability, user knowledge, and user attitudes [35]; and 1 evaluated user self-efficacy [34].

One study [34] reported having applied behavioral theories (self-efficacy theory was applied). The 4 remaining studies did not report using behavioral theories. However, the functionalities of the apps were investigated, and identifiable components of behavioral change strategies were discovered in every study, for example, self-monitoring, feedback, setting goals, and messages.

### Intervention Characteristics

The app characteristics are shown in Table 1. Each of the reviewed studies used a different app [31-35], with 2 apps commercially available (apps available to the public on an app store) [32,33] and 3 developed specifically for the study [31,34,35]. Among the 3 reviewed RCTs, the control groups in 2 RCTs received usual care [34,35], while the other control group received a mobile phone app to track food, without receiving feedback and motivational messages [33].

**Table 1:** App intervention characteristics.

App intervention type	App name (purpose)	App type	App functionalities
App + 1 other approach (communication with a coach by phone call to establish personalized DASH^a^ diet plans and feedback)	Noom (healthy weight loss and more)	Commercial^b^	Self-monitoring (BP^c^, weight, and PA^d^) Diet self-monitoring with a comprehensive and easily accessible nutrient database Educational information, goal setting, feedback, motivational messages, and reminder
App + 2 other approaches (motivation and feedback text message + DASH video and booklet)	Nutritionix (diet tracking)	Commercial	Diet self-monitoring with a comprehensive and easily accessible nutrient database
App + 1 other approach (communication with a coach by text message or email)	DASH mobile	Noncommercial^e^	Self-monitoring (BP, weight, daily diet, and PA). Educational information, feedback, motivational messages, goal setting, and communication with a coach by chat
App + 2 other approaches (phone call + text message)	DASH-related recommendations	Noncommercial	Educational information
App stand-alone	Blood Pressure Management Application (BPMAP)	Noncommercial	Self-monitoring (BP) Educational information, feedback, motivational messages, reminder, and DASH diet plan

^a^DASH: Dietary Approaches to Stop Hypertension.

^b^Smartphone app that is available on app stores.

^c^BP: blood pressure.

^d^PA: physical activity.

^e^Smartphone app that is not available on app stores.

### Outcomes

#### Effect on BP and Weight

All studies [31-35] examined the direct impact of DASH mobile app interventions on health outcomes in terms of BP, and 4 studies assessed weight loss [31,32,34,35].

Four studies reported a positive effect of the DASH diet app on both SBP and DBP [32-35], and 3 studies reported significant results [32,34,35]. In total, 3 studies reported significantly reduced weight loss (Tables 2 and 3) [32,34,35].

**Table 2:** Blood pressure and weight loss effects in randomized controlled trials.

Study and variable				Total length of the intervention		Blood pressure							Effect (blood pressure)			BMI (kg/m^2^)			Effect (BMI)
						SBP^a^ (mmHg)		DBP^b^ (mmHg)		Change in arterial pressure (mmHg)									
**Darabi et al [34]**				12 weeks						NR^c^		Positive^d^						Positive^d^	
	**Intervention (n=44), mean (SD)**																		
		Baseline			150.43 (10.19)		94.15 (7.69)							29.51 (2.89)					
		12 weeks			144.65 (10.36)		88.59 (8.34)							29.40 (2.91)					
	**Control (n=44), mean (SD)**																		
		Baseline			155.88 (16.81)		96.13 (8.41)							28.53 (2.57)					
		12 weeks			161.09 (17.46)		97.61 (7.27)							28.64 (2.62)					
**Bozorgi et al [35]**				24 weeks		NR		NR				Positive^d^						Positive^d^	
	**Intervention (n=60), mean (SD)**																		
		Baseline							108.9 (13.5)					29.7 (3.4)					
		24 weeks							94.8 (3.42)					28.6 (3.2)					
	**Control (n=60), mean (SD)**																		
		Baseline							114.9 (14.30)					28.5 (3.6)					
		24 weeks							100.1 (7.20)					28.4 (3.7)					
**Steinberg et al [33] (N=59)**				3 months						NR		Neutral^e^			NR			NR	
	Baseline for both groups, mean (SD)				122.9 (14.2)		80.2 (8.8)												
	Between group difference, mean (95% CI)				−2.8 (−1.8 to 7.4)		−3.6 (−0.2 to 7.3)												

^a^SBP: systolic blood pressure.

^b^DBP: diastolic blood pressure.

^c^NR: not reported.

^d^Blood pressure was significantly reduced by the app.

^e^Blood pressure was neutrally affected by the app.

**Table 3:** Blood pressure and weight loss effects in pre-post studies.

Study and variable		Total length of the intervention	Blood pressure		Effect (blood pressure)	BMI (kg/m^2^)	Effect (BMI)
			SBP^a^ (mmHg)	DBP^b^ (mmHg)			
**Weerahandi et al [31] (N=17)**		120 days			Neutral^c^		Neutral^c^
	Baseline, mean (SD)		138.6 (21.47)	86.9 (16.10)		33.6 (7.46)	
	120 days, mean (SD)		139.75 (15.85)	89.50 (13.85)		33.83 (7.64)	
**Toro-Ramos et al [32] (N=50)**		24 weeks			Positive^d^		Positive^d^
	Baseline, mean (SD)		130.93 (12.81)	83.03 (11.32)		33.60 (8.29)	
	Change from baseline to 24 weeks, mean (SD)		−5.98 (17.60)	−5.06 (11.89)		−1.21 (1.38)	

^a^SBP: systolic blood pressure.

^b^DBP: diastolic blood pressure.

^c^Blood pressure was neutrally affected by the app.

^d^Blood pressure was significantly reduced by the app.

#### DASH Diet Adherence

The 3 randomized studies [33,34,35] evaluated the effects of apps on dietary behavioral changes. The DASH score was used to evaluate adherence to DASH and was calculated using 9 [33,34] target nutrients. The sum of all nutrient goal values, with a maximum of 9, was used to calculate the DASH score. A value of 1 was assigned if the DASH target for a nutrient was met, 0.5 if the intermediate target was met, and 0 if no target was met [36]. Bozorgi et al [35] used a food frequency questionnaire to assess dietary change.

Three studies demonstrated that using a DASH app resulted in better adherence to the DASH diet and consequently lower BP (Table 4). Darabi et al [34] demonstrated that using a smartphone app to educate patients about the DASH diet and improve self-efficacy resulted in better adherence to the DASH diet, with significant differences between groups at the end of the trial. Bozorgi et al [35] evaluated the app’s impact on patient adherence to the DASH diet. They observed increased consumption of fruits, vegetables, and dairy in the intervention group compared with the control group. Moreover, the consumption of low-fat and low-salt diet plans increased by 1.7 and 1.5 points, respectively. Steinberg et al [33] compared dietary changes between women who used app-based diet tracking (control group) and those who used app-based diet tracking with feedback on DASH adherence through text messages (intervention group) over 3 months. They found that both groups’ DASH scores improved significantly after 3 months. A single-unit increase in the DASH score in the intervention group was linked to a 2.7 (95% CI 0.4-5) mmHg drop in SBP (*P*=.03) and a 1.3 (95% CI 1.0-3.6) mmHg drop in DBP (*P*=.26). In the control group, the association was a little weaker, with a single-unit increase in the DASH score linked to a 1.7 (95% CI 2.1-5.4) mmHg drop in SBP (*P*=.37) and a 1.8 (95% CI 0.8-4.4) mmHg drop in DBP (*P*=.26).

**Table 4:** Change in the Dietary Approaches to Stop Hypertension (DASH) adherence score.

Study (follow-up) and DASH^a^ score		Change in the DASH adherence score		
		Intervention group, mean (SD)	Control group, mean (SD)	Effect
**Darabi et al [34] (12 weeks)**				Positive^b^
	Baseline score	2.895 (0.457)	2.931 (0.534)	
	End of trial score	3.837 (0.761)	3.875 (0.699)	
**Bozorgi et al [35] (24 weeks)**				
	No assessment reported	NR^c^	NR	NR
**Steinberg et al [33] (12 weeks)**				Positive^b^
	Baseline score	2.2 (1.3)	2.3 (1.3)	
	End of trial score	3.1 (1.4)	3.1 (1.3)	

^a^DASH: Dietary Approaches to Stop Hypertension.

^b^DASH adherence was significantly increased by the app.

^c^NR: not reported.

#### App Usability and Acceptability, and User Engagement and Satisfaction

Four studies assessed user engagement [31-33,35], 2 evaluated user satisfaction [33,35], and 1 evaluated acceptance [31]. All focused on the patients’ perspectives, and 1 study also assessed patients’ knowledge and app usability [35].

User engagement was assessed by logging food intake, BP, weight, and step count. Chats, phone calls, and text messages were also incorporated [31-33,35]*.* Generally, participants’ use of the apps to record food, BP, and weight was high.

In the 2 studies that evaluated user satisfaction, participants were very accepting of the use of apps [33,35]. In the study by Steinberg et al [33], participants reported that the app was easy to use, and that they used it frequently and would recommend it to friends [33]. They also reported that the DASH score was helpful and motivational, and that the timing of the text messages was convenient and helped them achieve their goals [33].

In the study conducted by Bozorgi et al [35], the results suggested that usability was good.

### Quality Appraisal of Studies

**Figure 2:**
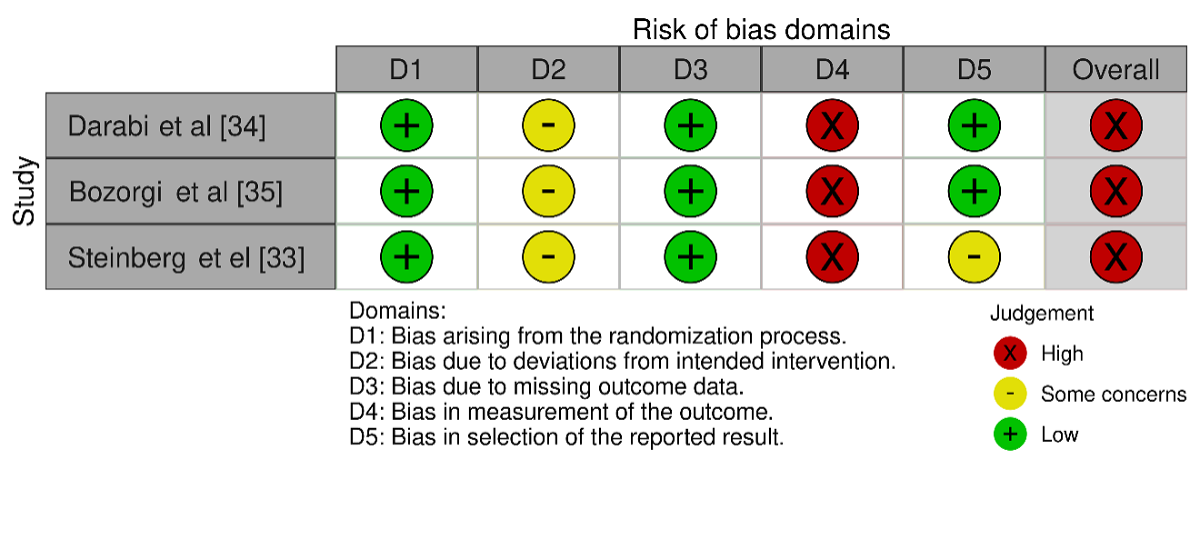
Summary of the risk of bias assessment using the Cochrane Collaboration’s Risk of Bias tool.

All included RCTs used an appropriate random allocation sequence for randomization. The allocation sequences were concealed by all studies until the participants were enrolled. Therefore, all studies had low bias risk due to randomization (Figure 2).

The staff in studies testing the DASH diet smartphone app and the participants were aware of the assigned interventions in 3 and 2 studies, respectively. In all studies, a suitable analysis was used to estimate the effect of the assigned intervention (intention-to-treat or modified intention-to-treat analysis). Accordingly, the risk of bias due to deviations from intended interventions had “some concerns” in all studies.

In all studies, outcome data were available for most or all participants. The “missing outcome data” domain was deemed to have low risk of bias in all studies (Figure 2).

All included studies evaluated the outcome of interest (ie, BP level and DASH diet adherence) using appropriate measures and used methods that were comparable between intervention groups. However, in all studies, the assessor of the outcome was not blinded. For this reason, all studies were rated as having high risk of bias in the “measuring the outcome” domain (Figure 2).

The prespecified analysis plan (eg, protocol) was published in 2 studies. Therefore, 2 studies were considered to have low risk of bias due to selection of reported results (Figure 2).

All studies were judged to have high risk of bias in the last domain, “overall bias,” because they had a high risk in at least one domain.

Of the 2 pre-post studies, 1 was of poor quality [31] due to the study’s design (pilot study, small sample size, and lack of power analysis). Moreover, there was some missing information that affected study validity. The other study [32] was deemed to be of fair quality because it had a good sample size, and a clear method was used. Quality assessment results are reported in Multimedia Appendix 4.

## Discussion

### Principal Findings

This systematic review aimed to synthesize evidence on the effectiveness of DASH smartphone apps that support self-management in order to improve DASH diet adherence and consequently reduce BP. It also aimed to examine satisfaction, acceptability, engagement, and usability of DASH smartphone apps. Our review highlighted weak emerging evidence of a positive effect of using DASH smartphone apps. However, the evidence is inconclusive because some studies on the topic were of low quality due to the fact that blinding of participants and assessors was not implemented, and the study protocol was not published. Furthermore, 1 of the 3 studied RCTs was unpublished, that is, the manuscript is under review. Therefore, the data do not allow firm conclusions about the effectiveness of DASH smartphone apps to increase DASH diet adherence and lower BP.

This review indicates that a DASH mobile app that engages patients and encourages self-management of the DASH diet may be helpful in improving adherence to the DASH diet. The findings are in line with the findings of other systematic reviews that involved chronic kidney disease dietary mobile app interventions for changing user dietary behavior, which illustrated that the use of nutritional apps enhanced adherence to sodium reduction, protein intake, caloric intake, and fluid dietary limitations [37,38]. Our results showed that using a DASH smartphone app may improve DASH diet adherence and consequently reduce BP and body weight [32,34,35]. This is consistent with systematic reviews that have focused on traditional interventions, which showed that adherence to the DASH diet significantly reduces SBP, DBP, and body weight [18,39].

In this review, all apps had some similar functionalities; 3 out of the 5 apps combined 3 functionalities, including educational information, feedback, and messages (reminder or motivation), with other functions. We could not determine the most effective functionalities because there was no clear difference in the results between apps with different functionalities [23,40]. In this review, we found no difference between commercial and noncommercial apps in terms of their characteristics.

Interventions involved the mobile app alone or in combination with other communication tools, such as phone calls, chats through the app itself, or text messaging. It was not possible to determine from the results whether combining the app with other modalities increased effectiveness. However, Schoeppe et al [41] found that apps were most successful when combined with other tools rather than used as a stand-alone intervention.

The findings with regard to usability and feasibility are in line with studies assessing dietary smartphone apps for changing the behavior of chronic kidney disease patients [37], which also found that the apps were useable and feasible. Studies assessing the acceptance and usability of mobile apps for chronic disease management support our results regarding acceptance [40,42].

After examining the risk of bias of the included studies, the findings of this review should be treated with caution because several studies had high risk of bias. Three RCTs and 2 pre-post pilot studies were included. Four out of the 5 studies had methodological issues. These difficulties arose from potential biases in all RCTs because blinding of participants and assessors was not implemented, the study protocol was not published [33], or the study duration was short [31-35]. Due to the nature of using apps, blinding of subjects was not possible across interventions. One of the 3 RCTs is still under review [34]. All RCTs used an appropriate random allocation sequence for randomization and concealed the allocation sequence [33-35]. The outcome data were available for most or all participants [33-35]. One pre-post pilot study had limitations that included small sample size, short duration, and missing information [31].

### Strengths and Limitations of This Review

The studies included in this review have some limitations. First, 4 included studies were evaluated as “low quality,” implying that unreliable outcomes were possible. These factors, together with heterogeneous outcomes and the methods used to quantify them, make drawing generalizable conclusions difficult. Owing to the variability in study design, a meta-analysis was not possible. Second, 1 study was under review, and low-quality studies were considered because more recent findings are often helpful. Finally, in the included studies, the socioeconomic characteristics of participants were rarely reported; nevertheless, when they were reported, they revealed a high educational level, thus further limiting the generalizability of the results.

Additionally, this review has certain limitations. First, few studies exploring the use of smartphone apps to enhance DASH diet adherence could be found, even though the authors established a comprehensive search strategy for the 5 main databases and manually reviewed the reference lists of each full-text article to identify potentially relevant research for inclusion in this systematic review. Second, due to the low number of RCTs, we were unable to evaluate the effectiveness of DASH smartphone apps. Third, studies written in languages other than English were excluded, increasing the chance of relevant research being missed.

Despite these limitations, to our knowledge, this is the first systematic review investigating the effectiveness of using smartphone apps for patient adherence to the DASH diet, which is known to lower BP, and assessing user satisfaction and app acceptance. This review also highlights the crucial issue of the lack of high-quality research in this field, and thus, this review could help improve future research on the use of DASH smartphone apps by people with hypertension.

### Future Directions

In general, the methodological quality of the research included in this study was poor. This suggests that future studies should include a sufficient number of participants and a sufficiently long duration, and should ensure blinding of assessors and low attrition rates. It would also be beneficial to conduct a well-designed RCT with multiple arms using apps with different combinations of functionalities to identify the most effective combinations. The results of this review are applicable to short-term app use because most interventions lasted between 3 and 6 months. Longer-term studies are needed to integrate smartphone apps into people’s daily routines and assess their usefulness for long-term DASH diet adherence. It is also essential to evaluate and understand users’ acceptance of and satisfaction with these apps. Most studies included in this review evaluated DASH diet adherence by calculating the DASH score based on a food recall questionnaire that may be impacted by inaccurate reporting by participants [33-35]. Future studies should incorporate objective measures, such as urinary excretion, to measure dietary adherence to DASH [43].

### Conclusion

This review identified 5 studies including a total of 334 participants. Use of smartphone apps to increase DASH diet adherence and reduce BP in hypertensive patients is clearly in the early stages of development. However, the fact that studies were found in 2 different countries (using 5 smartphone apps with similar functionalities) and that all of them were published in the last 4 years indicates that the research community is now taking interest in the DASH diet. All the apps seemed to be accepted and easy to use. Although it is impossible to draw firm conclusions from the current evidence, the studies indicated positive trends, suggesting that DASH smartphone apps could be useful tools to increase DASH diet adherence and reduce BP. Further research is needed that can provide higher-quality evidence to determine the effectiveness of DASH smartphone apps to improve adherence to the DASH diet and correspondingly lower BP.

## Appendix

Multimedia Appendix 1 Search strategy.

Multimedia Appendix 2 PRISMA (Preferred Reporting Items for Systematic Reviews and Meta-Analyses) checklist.

Multimedia Appendix 3 Summary of study characteristics.

Multimedia Appendix 4 Quality criteria checklist of articles included in the systematic review.

## Abbreviations

BP: blood pressure
DASH: Dietary Approaches to Stop Hypertension
DBP: diastolic blood pressure
NHLBI: National Heart, Lung, and Blood Institute
RCT: randomized controlled trial
SBP: systolic blood pressure
