# Supplementary material for: The Use of Dietary Approaches to Stop Hypertension (DASH) Mobile Apps for Supporting a Healthy Diet and Controlling Hypertension in Adults: Systematic Review
Source: JMIR Cardio. 2022 Nov 2;6(2):e35876. doi: 10.2196/35876 (PMC9669886; doi:10.2196/35876)
Supplement: Multimedia Appendix 1 [file cardio_v6i2e35876_app1.docx]

Multimedia Appendix 1: Search strategy.

| ***Scopus 2008- February 2021*** | | |
| --- | --- | --- |
| ***No.*** | ***Query*** | ***Results*** |
| *1* | (TITLE-ABS-KEY ("Dietary Approach* to Stop Hypertension") OR TITLE-ABS-KEY ("DASH diet")) | *1712* |
| *2* | ( TITLE-ABS-KEY ( "mobile application" )  OR  TITLE-ABS-KEY ( "smartphone app*" )  OR  TITLE-ABS-KEY ( "smartphone" )  OR  TITLE-ABS-KEY ( "Cell phone" )  OR  TITLE-ABS-KEY ( "digital technology" )  OR  TITLE-ABS-KEY ( "mHealth" )  OR  TITLE-ABS-KEY ( "mobile health" )  OR  TITLE-ABS-KEY ( "mobile phone application" )  OR  TITLE-ABS-KEY ( "Mobile adj3 app*" )  OR  TITLE-ABS-KEY ( "*medical informatics applications" )  OR  TITLE-ABS-KEY ( "Ehealth" )  OR  TITLE-ABS-KEY ( "e?health" )  OR  TITLE-ABS-KEY ( "m?health" )  OR  TITLE-ABS-KEY ( "window adj3 phone" )  OR  TITLE-ABS-KEY ( "Apps" )  OR  TITLE-ABS-KEY ( "Iphone" )  OR  TITLE-ABS-KEY ( "software adj3 app*" )  OR  TITLE-ABS-KEY ( "mobile adj3 app*" )  OR  TITLE-ABS-KEY ( "mobile adj3 software" )  OR  TITLE-ABS-KEY ( "telehealth" )  OR  TITLE-ABS-KEY ( "Computer" ) ) | *5,197,012* |
| *#1 AND #2* | [( ( TITLE-ABS-KEY ( "mobile application" )  OR  TITLE-ABS-KEY ( "earphone app*" )  OR  TITLE-ABS-KEY ( "earphone" )  OR  TITLE-ABS-KEY ( "Cell phone" )  OR  TITLE-ABS-KEY ( "digital technology" )  OR  TITLE-ABS-KEY ( "health" )  OR  TITLE-ABS-KEY ( "mobile health" )  OR  TITLE-ABS-KEY ( "mobile phone application" )  OR  TITLE-ABS-KEY ( "mobile adj app*" )  OR  TITLE-ABS-KEY ( "*medical informatics applications" )  OR  TITLE-ABS-KEY ( "health" )  OR  TITLE-ABS-KEY ( "e?health" )  OR  TITLE-ABS-KEY ( "m?health" )  OR  TITLE-ABS-KEY ( "window adj phone" )  OR  TITLE-ABS-KEY ( "Apps" )  OR  TITLE-ABS-KEY ( "phone" )  OR  TITLE-ABS-KEY ( "software adj app*" )  OR  TITLE-ABS-KEY ( "mobile adj app*" )  OR  TITLE-ABS-KEY ( "mobile adj software" )  OR  TITLE-ABS-KEY ( "telhealth" )  OR  TITLE-ABS-KEY ( "Computer" ) ) )  AND  ( ( TITLE-ABS-KEY ( "Dietary Approach* to Stop Hypertension" )  OR  TITLE-ABS-KEY ( "DASH diet" ) ) )](https://www.scopus.com/results/documentSpellSuggest.uri?sort=plf-f&src=s&mltEid=&mltAll=t&sid=f56b08eabc067ee41000a4f691f53127&sot=comb&sdt=comb&sl=885&s=%28+%28+TITLE-ABS-KEY+%28+%22mobile+application%22+%29+OR+TITLE-ABS-KEY+%28+%22earphone+app*%22+%29+OR+TITLE-ABS-KEY+%28+%22earphone%22+%29+OR+TITLE-ABS-KEY+%28+%22Cell+phone%22+%29+OR+TITLE-ABS-KEY+%28+%22digital+technology%22+%29+OR+TITLE-ABS-KEY+%28+%22health%22+%29+OR+TITLE-ABS-KEY+%28+%22mobile+health%22+%29+OR+TITLE-ABS-KEY+%28+%22mobile+phone+application%22+%29+OR+TITLE-ABS-KEY+%28+%22mobile+adj+app*%22+%29+OR+TITLE-ABS-KEY+%28+%22*medical+informatics+applications%22+%29+OR+TITLE-ABS-KEY+%28+%22health%22+%29+OR+TITLE-ABS-KEY+%28+%22e%3fhealth%22+%29+OR+TITLE-ABS-KEY+%28+%22m%3fhealth%22+%29+OR+TITLE-ABS-KEY+%28+%22window+adj+phone%22+%29+OR+TITLE-ABS-KEY+%28+%22Apps%22+%29+OR+TITLE-ABS-KEY+%28+%22phone%22+%29+OR+TITLE-ABS-KEY+%28+%22software+adj+app*%22+%29+OR+TITLE-ABS-KEY+%28+%22mobile+adj+app*%22+%29+OR+TITLE-ABS-KEY+%28+%22mobile+adj+software%22+%29+OR+TITLE-ABS-KEY+%28+%22telhealth%22+%29+OR+TITLE-ABS-KEY+%28+%22Computer%22+%29+%29+%29+AND+%28+%28+TITLE-ABS-KEY+%28+%22Dietary+Approach*+to+Stop+Hypertension%22+%29+OR+TITLE-ABS-KEY+%28+%22DASH+diet%22+%29+%29+%29&origin=resultslist) | *35* |

| ***Cochrane Library 2008- February 2021*** | | |
| --- | --- | --- |
| ***No.*** | ***Query*** | ***Results*** |
| #1 | MeSH descriptor: [Dietary Approach* to Stop Hypertension ] explode all trees | 511 |
| #2 | MeSH descriptor: [DASH diet ] explode all trees | 623 |
| #3 | #1 OR #2 | 723 |
| #4 | mobile application | 4147 |
| #5 | smartphone app* | 4225 |
| #6 | smartphone | 5213 |
| #7 | Cell phone | 2061 |
| #8 | digital technology | 1641 |
| #9 | mHealth | 2076 |
| #10 | mobile health | 7738 |
| #11 | mobile phone application | 1194 |
| #12 | Mobile adj3 app* | 241 |
| #13 | *medical informatics applications | 178 |
| #14 | Ehealth | 1645 |
| #15 | e?health | 1648 |
| #16 | m?health | 2104 |
| #17 | window adj3 phone 6 | 24 |
| #18 | Apps | 3385 |
| #19 | Iphone | 304 |
| #20 | software adj3 app* | 1976 |
| #21 | mobile adj3 app* | 241 |
| #22 | mobile adj3 software | 181 |
| #23 | telehealth | 2692 |
| #24 | Computer | 49656 |
| #25 | #4 OR #5 OR #6 OR #7 OR #8 OR #9 OR #10 OR #11 OR #12 OR #13 OR #14 OR #15 OR #16 OR #17 OR #18 OR #19 OR #20 OR #21 OR #22 OR #23 OR #24 | 67591 |
| #26 | #3 AND #25 | 84 |

| **Embase *2008-February 2021*** | | |  |
| --- | --- | --- | --- |
| ***No.*** | ***Query*** | ***Results*** | **Type** |
| 1 | Dietary Approach* to Stop Hypertension.mp. | 1440 | Advanced |
| 2 | DASH diet.mp. | 1565 | Advanced |
| 3 | 1 OR 2 | 2037 | Advanced |
| 4 | mobile application.mp. | 18814 | Advanced |
| 5 | smartphone app*.mp. | 6881 | Advanced |
| 6 | smartphone.mp. | 27114 | Advanced |
| 7 | Cell phone.mp.or mobile phone | 22121 | Advanced |
| 8 | digital technology.mp. | 3928 | Advanced |
| 9 | mHealth.mp. | 6976 | Advanced |
| 10 | mobile health.mp. | 8576 | Advanced |
| 11 | mobile phone application.mp. | 495 | Advanced |
| 12 | (Mobile adj3 app*).mp. | 26001 | Advanced |
| 13 | *medical informatics applications/ | 10915 | Advanced |
| 14 | Ehealth.mp.or telehealth/ | 17316 | Advanced |
| 15 | e?health.mp. | 6012 | Advanced |
| 16 | m?health.mp. | 7028 | Advanced |
| 17 | (window adj3 phone).mp. | 7 | Advanced |
| 18 | Apps.mp. | 11249 | Advanced |
| 19 | Iphone.mp. | 2213 | Advanced |
| 20 | (software adj3 app*).mp. | 10456 | Advanced |
| 21 | (mobile adj3 app*).mp. | 26001 | Advanced |
| 22 | (mobile adj3 software).mp. | 384 | Advanced |
| 23 | Computer.mp. | 1444116 | Advanced |
| 24 | 4 or 5 or 6 or 7 or 8 or 9 or 10 or 11 or 12 or 13 or 14 or 15 or 16 or 17 or 18 or 19 or 20 or 21 or 22 or 23 or 24 | 1532211 | Advanced |
| 25 | #3 AND #25 | 30 |  |

| ***Web of Science 2008- February 2021*** | | |
| --- | --- | --- |
| ***No.*** | ***Query*** | ***Results*** |
| 1 | Dietary Approach* to Stop Hypertension OR DASH diet | 5,647 |
| 2 | mobile application or smartphone app* or Cell phone or digital technology or mobile health or mobile phone application or mHealth or Mobile adj3 app* or *medical informatics applications or Ehealth or e?health or m?health or Apps or Iphone or mobile adj3 app* or telehealth or Computer or software adj3 app* or window adj3 phone | 284,060 |
| 3 | #1 AND #2 | 19 |

| ***CINAHL 2008- February2021*** | | |
| --- | --- | --- |
| ***No.*** | ***Query*** | ***Results*** |
| S1 | Dietary Approach* to Stop Hypertension OR dash diet | 1180 |
| S2 | ( mobile application or smartphone app* or Cell phone or digital technology or mobile health or mobile phone application or mHealth ) OR ( Mobile adj3 app* or *medical informatics applications or Ehealth or e?health or m?health or Apps or Iphone or mobile adj3 app* or telehealth ) OR ( Computer or software adj3 app* or window adj3 phone ) | 204,968 |
| S3 | (( mobile application or smartphone app* or Cell phone or digital technology or mobile health or mobile phone application or mHealth ) OR ( Mobile adj3 app* or *medical informatics applications or Ehealth or e?health or m?health or Apps or Iphone or mobile adj3 app* or telehealth ) OR ( Computer or software adj3 app* or window adj3 phone )) AND (S1 AND S2) | 15 |
