# Supplementary material for: The Use of Dietary Approaches to Stop Hypertension (DASH) Mobile Apps for Supporting a Healthy Diet and Controlling Hypertension in Adults: Systematic Review
Source: JMIR Cardio. 2022 Nov 2;6(2):e35876. doi: 10.2196/35876 (PMC9669886; doi:10.2196/35876)
Supplement: Multimedia Appendix 3 [file cardio_v6i2e35876_app3.docx]

Multimedia Appendix 3: Summary of study characteristics

| **First Author, Year and Country** | **Study Tittle** | **Study Characteristics** | | **Input** | | **Intervention** | | | **outcome** | |
| --- | --- | --- | --- | --- | --- | --- | --- | --- | --- | --- |
|  |  | **Participants (Sample Size, Mean Age)** | **Study Method, Duration** | **^e^mode** | **^g^ content** | **mode** | **content** | **Theory** | **mode** | **content** |
| Weerahandi et al (2020) USA [31] | A Mobile Health Coaching Intervention for Controlling Hypertension: Single-Arm Pilot Pre-Post Study | N=17  age=18-65 prehypertension and stage 1 hypertension | Pilot study  Pre-post evaluation  120 days | Text | Daily diet, BP, weight Step, goal, chat | In-App log, In-App chat with coach, Feedback by phone call, SMS, email | Personalized feedback about diet, number of steps, goal educational information | ^f^ NR | Self- reported | **^c^ HS**: BP,  weight, BMI, heart rate,  **^b^ BC**: PA  **Engagement and Acceptability** |
| Darabi et al (2020) Iran [34] | The effectiveness of a mobile phone education- based on self-efficacy and DASH diet among patient with high blood pressure: a randomized controlled trial | N=88  age= 30-69 hypertension | RCT  12 weeks | -- | -- | App + patient’s compliance monitored by phone call, weekly text message | DASH diet recommendation based on self-efficacy | self- efficacy | Self- reported  Questionnaire and clinical outcome measured by research team | **HS:** BP, weight and height  **BC:** self- efficacy +PA |
| Toro-Ramos et al (2017) USA [32] | Efficacy of a mobile hypertension prevention delivery platform with human coaching | N=50  age =18-75 prehypertension, hypertension | Pilot study per-post evaluation  24 weeks | Text | Daily diet, BP, weight, and PA, | In -App log,  In – App communication by application message, biweekly phone call | Individual plan and goal with coach, educational information | Cognitive behavioural therapy, motivational interviewing | Self- reported | **HS:** BP**,** weight  **Engagement** |
| **First Author, Year and Country** | **Study Tittle** | **Study Characteristics** | | **Input** | | **Intervention** | | | **outcome** | |
|  |  | **Participants (Sample Size, Mean Age)** | **Study Method, Duration** | **mode** | **content** | **mode** | **content** | **Theory** | **mode** | **content** |
| Bozorgi et al (2021)  [35] Iran | The Effect of the Mobile ‘Blood Pressure Management Application’ on Hypertension Self-Management Enhancement: A Randomized Controlled Trial | N=120, age= 30-60 primary hypertension | RCT  24 weeks | Text | Daily BP | In -App log | Received feedback, reminder time for drug, visit date and BP measurement, DASH diet plan, send notification to the family, individual motivation messages | NR | The adherence to treatment measured by the Hill-Bone Scale, the clinical outcome measured by physician. The questionnaire was completed by trained researcher. | **HS**: BP, weight,  **BC:** adherence to treatment, and PA  **^a^DBC:** adherence to healthy diet ^d^ DASH  **Satisfaction, Knowledge, attitudes** |
| Steinberg et al (2020) USA [33] | Feasibility of a Digital Health Intervention to Improve Diet Quality Among Women with High Blood Pressure: Randomized Controlled Feasibility Trial | N=59, age= 21-70 hypertension | RCT  3month | Text | Daily diet | In- app log, SMS daily or weekly feedback with motivational message | Tailored feedback with  DASH score, motivational message, educational video, tips for certain dietary change | NR | Self- reported questionnaires clinical outcome measured by research team | **HS:** BP  **DBC:**  adherence to healthy diet (DASH)  **Engagement, Satisfaction** |

^a^ DBC: Dietary behaviour change, ^b^ BC: Behaviour change, ^c^ HS: Health status, ^d^ Dietary Approach to Stop Hypertension

^e^ Mode: How to enter data in the app or how to deliver the intervention.  ^f^ NR: Not reported

^g^ Content: the information that intervention gives or requires from user
