# Supplementary material for: The Use of Dietary Approaches to Stop Hypertension (DASH) Mobile Apps for Supporting a Healthy Diet and Controlling Hypertension in Adults: Systematic Review
Source: JMIR Cardio. 2022 Nov 2;6(2):e35876. doi: 10.2196/35876 (PMC9669886; doi:10.2196/35876)
Supplement: Multimedia Appendix 4 [file cardio_v6i2e35876_app4.docx]

Multimedia Appendix 4: Quality criteria checklist of articles included in the systematic review.

| **Author(s) (year** | **Quality rating** | **Validity Questions ^a^** | | | | | | | | | | | | **Comments /study limitations** |
| --- | --- | --- | --- | --- | --- | --- | --- | --- | --- | --- | --- | --- | --- | --- |
|  |  | **1** | **2** | **3** | **4** | **5** | **6** | **7** | **8** | **9** | **10** | **11** | **12** |  |
| Weerahandi et al (2020) | Poor quality | Y | Y | Y | Y | N | Y | N | NR | Y | Y | CD | NA | Despite having a good attrition rate. Bias was generated due to the study's design (pilot study, small sample size, lack of power analysis). Some missing information that affected study validity, it was not well-reported. |
| Toro-Ramos et al (2017) | Fair quality | Y | Y | Y | Y | Y | Y | Y | NR | N | Y | N | NA | Some have potential for titration bias, but it has a good sample size, a clear method was used and procedures well-reported. |

N = no; Y = yes; CD = cannot determine; NA = not applicable; NR = not reported

**Validity Questions ^a^**

1. Was the study question or objective clearly stated?

2.Were eligibility/selection criteria for the study population prespecified and clearly described?

3.Were the participants in the study representative of those who would be eligible for the test/service/intervention in the general or clinical population of interest?

4.Were all eligible participants that met the prespecified entry criteria enrolled?

5. Was the sample size sufficiently large to provide confidence in the findings?

6.Was the test/service/intervention clearly described and delivered consistently across the study population?

7.Were the outcome measures prespecified, clearly defined, valid, reliable, and assessed consistently across all study participants?

8. Were the people assessing the outcomes blinded to the participants' exposures/interventions?

9. Was the loss to follow-up after baseline 20% or less? Were those lost to follow-up accounted for in the analysis?

10. Did the statistical methods examine changes in outcome measures from before to after the intervention? Were statistical tests done that provided p values for the pre-to-post changes?

11. Were outcome measures of interest taken multiple times before the intervention and multiple times after the intervention (i.e., did they use an interrupted time-series design)?

12. If the intervention was conducted at a group level (e.g., a whole hospital, a community, etc.) did the statistical analysis take into account the use of individual-level data to determine effects at the group level?
